# Cross Sectional Study on Exposure to BPA and Phthalates and Semen Parameters in Men Attending a Fertility Center

**DOI:** 10.3390/ijerph17020489

**Published:** 2020-01-13

**Authors:** Lidia Caporossi, Alessandra Alteri, Giovanni Campo, Enrico Paci, Giovanna Tranfo, Silvia Capanna, Enrico Papaleo, Daniela Pigini, Paola Viganò, Bruno Papaleo

**Affiliations:** 1 National Institute of Insurance against Accidents at Work-Department of Occupational and Environmental Medicine, Epidemiology and Hygiene, 00078 Monte Porzio Catone, Italy; e.paci@inail.it (E.P.); g.tranfo@inail.it (G.T.); s.capanna@inail.it (S.C.); d.pigini@inail.it (D.P.); b.papaleo@inail.it (B.P.); 2 Unit of Obstetrics and Gynecology, San Raffaele Scientific Institute, 20132 Milan, Italy; alteri.alessandra@hsr.it (A.A.); campo.giovanni@hsr.it (G.C.); papaleo.enrico@hsr.it (E.P.); 3 Reproductive Sciences Laboratory, Division of Genetics and Cell Biology, San Raffaele Scientific Institute, 20132 Milan, Italy; vigano.paola@hsr.it

**Keywords:** semen, phthalate, bisphenol A, reproduction

## Abstract

Among the possible risk factors for male reproduction, exposure to phthalates and alkylphenols is widely documented. This study evaluated the possible association between chemical exposure and the quality of the seminal fluid of 105 subjects in a fertility clinic. The urinary levels of seven phthalate metabolites (monoethylphthalate, MEP; monobenzylphthalate, MBzP; mono *n*-butylphthalate, MnBP; mono-(2-ethylhexyl) phthalate, MEHP; mono(2-ethyl-5-hydroxyhexyl) phthalate, MEHHP; mono-*n*-octylphthalate, MnOP; mono-isononylphthalate, MiNP) and bisphenol A (BPA), were analysed by high performance liquid chromatography/tandem mass spectrometry HPLC/MS/MS. The regression analysis showed that the semen volume was positively associated with MnBP, MnOP and BPA levels while was negatively associated with MiNP levels. The sperm concentration had a significant inverse relationship with MEP levels. A negative association was found between the use of plastic containers for food storage (*p* = 0.037) and semen volume (3.06 vs. 2.30 mL as average values, never vs daily). A significant positive correlation emerged (*p* < 0.005) between the consumption of canned food and the levels of BPA (2.81 vs. 0.14 µg/g creat as average values, daily vs. never) and between the use of perfumes and levels of MEP (389.86 vs. 48.68 µg/g creat, as average values, daily vs. never). No further statistically significant associations were found, even considering the working activity. Some evidence emerged about the possible link between exposure and seminal fluid quality: further case/control or prospective studies will allow us to confirm this causality hypothesis.

## 1. Introduction

Subfertility is a serious health and social problem which affects about 10% of the population [1]. Males constitute 25–30% of all fertility problem cases [2]. In the last decades some evidence has shown that semen quality, expressed in terms of sperm count, has declined [3,4] although this finding is still debated [5,6,7,8]. The exposure to chemicals with potential toxic effects on reproduction seems to be involved in the decline of semen quality. A recent publication [9] studying the temporal trends between 2000 and 2017 for semen parameters in the USA showed that the sperm concentration and count declined by 2.62% and 3.12% per year, respectively, corresponding to an overall decline of 37% and 42% over 17 years. More specifically, the researchers analysed urinary levels of some environmental pollutants, and they found a link with phthalates exposure. 

Phthalates [10] are industrial plasticizers with numerous applications in different products; humans are exposed by different routes to phthalates, which are rapidly metabolized to their monoesters. Some of them can be oxidized or conjugated with glucuronic acid before being excreted in urine [11,12]. Several epidemiological studies have focused on the male health effects of phthalate exposure [13,14] and documented an association between phthalates metabolites levels, particularly mono *n*-butylphthalate (MnBP) and mono-(2-ethylhexyl) phthalate (MEHP) [15,16,17] and lowered sperm concentration, lower density and decreased motility, even if some controversies exist [18].

In particular, for MEHP levels a negative association with sperm motility was observed. Other authors found as well an inverse association of monobenzylphthalate (MBzP) levels with sperm motility [19].

Moreover, an interesting study [20] found a reduced semen quality, even at phthalate levels below the US EPA reference doses. Particularly monoethylphthalate (MEP) concentration was associated with decreased sperm concentration (−5.3%), total sperm count (−5.7%) and motility (−2.6%). Wang et al. [21] confirmed these data showing that higher urinary MEP levels were significantly associated with a decreasing percentage of normal sperm morphology, with mediation of serum free thyroxine. Other authors conversely supported a lower sperm motility, with mediation by DNA methylation, linked to phthalate exposure [22].

Furthermore, published surveys, aiming to understand a possible correlation between phthalate exposure and male reproductive function [23], have shown an inverse association between urinary levels of phthalates metabolites and hormonal levels. Indeed, MEHP, MnBP and mono-isobutylphthalate (MiBP) levels were found to be inversely related with total and free testosterone, free androgen index and luteinizing hormone, while a positive association was reported with DNA fragmentation index. Overall phthalate exposure was associated also with altered reproductive hormones concentrations, linked to some extent to a lower semen quality [24].

To understand how phthalates could affect male reproduction, numerous in vitro and in vivo studies were carried out. The diethylhexylphthalate (DEHP) exposure induced, in rats, testicular toxicity through oxidative stress injury, DEHP levels were linked to the decrement of testosterone levels and upregulated the expression of the nuclear factor-erythroid 2 related factor (Nrf2) [25]. Di-*n-*butyl phthalate (DnBP) can lead to testicular damage, it impaired the normal structure of testicular tissue, increased sperm abnormality, decrease the viability of Sertoli cells through the activation of extracellular signal regulated kinase 1/2 (ERK1/2) and c-Jun amino terminal kinase (JNK) pathways [26]. To use as a model for human testicular dysgenesis syndrome, pregnant rats were exposed to DnBP and a suppression of intratesticular testosterone, a focal aggregation of Leyding cells and ectopic Sertoli cells were obtained [27]. Also, the possible molecular mechanism of MnBP was investigated in vitro and even at low dose, an increased Sertoli cells proliferation emerged [28].

Among other chemicals, evidence from previous studies showed that also bisphenol A (BPA), a precursor to important plastics like polycarbonates and epoxy resins, could interact with the endocrine system [29,30] and semen quality as well [31,32] even if the present evidence is controversial.

According to Lassen et al. [33] the association between BPA levels and reproductive hormones would support an anti-androgenic effect with deleterious consequences on motile spermatozoa (−6.7 %, confidence interval–CI −11.76/1.63 95%), data confirmed also by Radwan et al. [34]. 

BPA induced, in in vivo studies, testicular dysfunction and alteration of Sertoli cell viability [35], testicular apoptosis in male rats [36]; after BPA exposure spermatogenic cells were disorganized and degenerated, spermatids had fragmented pyknotic nuclei and a significant decrease in the rate of proliferation of germ cells was present [37].

In an epidemiological survey on 215 young health adults, the urinary BPA levels were significantly and inversely associated with sperm concentration and total sperm count [38]. In recent surveys [39,40] sperm concentration, morphology and motility were negatively associated with BPA urinary levels. Other authors [41] underlined a negative correlation between BPA levels and semen DNA fragmentation but not with other semen quality parameters.

Moreover, emerging evidence suggests that phthalates and BPA can play roles in the development of metabolic pathologies, from obesity to type 2 diabetes [42,43], in particular promoting adipogenesis [44] via peroxisome proliferator-activated receptor gamma [45]. In studies performed on mixtures of different chemicals, a more defined role for MnBP [46], MEP and BPA [47] emerged. Particularly for BPA the scientific debate was very broad [48,49], with controversial opinions, especially for epidemiological evidences, even if significant associations between BPA exposure and general abdominal obesity and metabolic diseases were substantial [50,51]. Obesity induces a reduced quality of seminal fluid (reduction in sperm count and motility) and the effects at the cellular level, particularly on testicular Leyding cells, were documented, the simultaneous exposure to phthalates strengthening this pathological effect [52].

Therefore, the aim of the present study was to participate in the scientific debate about the possible correlation between exposure to BPA and phthalates and human semen quality, with a cross-sectional study on men, focusing also on possible risk source in working and life environment.

## 2. Materials and Methods

### 2.1. Study Design and Participants

Participants were male partners of sub-fertile couples who attended the Reproductive Center of San Raffaele Institute in Milan (Italy), with unknown fertility status. Because the causes of infertility in a couple could be related both to male and/or female subject, the participants in the present survey included both men with fertility problems (abnormal sperm quality parameters) and healthy ones.

The Ethics Committee of the San Raffaele Institute in Milan approved the research protocol (identification code 73/INT/2017). From October 2017 to December 2018, 155 subjects were enrolled and each participant gave a written informed consent before participation.

The exclusion criteria were: self-reported endocrine diseases (e.g., adrenal disorders with clear etiology), self-reported clinical conditions that could affect semen quality (e.g., testis injury, vasectomy, epididymitis, orchiditis, varicocele), inability to produce a useful semen sample (e.g., azoospermia), missed urine samples, creatinuria out of the World Health Organization (WHO) range of normality for biological monitoring [53]. Using these criteria, the starting population was reduced to 105 men.

Furthermore, an ad hoc questionnaire was set up for the collection of anamnestic information and life and working habits. Therefore, every enrolled subject completed the questionnaire under the guidance of well-trained investigators. The collected data included: life habits like smoking or alcohol drinking, diet, use of plastic containers to store fat foods, occupational activity, clinical history (with specific focus on endocrine problems). The working activities were classified in relation to the possible presence of risk factors for male reproduction.

### 2.2. Urine Collection and Metabolite Analysis

The analytical reference standards of MEP, MBzP, MnBP, MEHP, mono(2-ethyl-5-hydroxyhexyl) phthalate (MEHHP), mono-*n*-octylphthalate (MnOP) and MiNP were purchased from Cambridge Isotopes Laboratories Inc. (Cambridge, MA, USA).

BPA and a deuterium labeled bisphenol d_6_ (BPA d_6_) internal standard were obtained from Sigma Aldrich (Saint Louis, MO, USA). The internal standards ^13^C-4 monobenzylphthalate (MBzP-^13^C) (ring-1,2-^13^C_2_, dicarboxyl-^13^C_2_, 99.9% and ^13^C-4 monoethylphthalate (MEP-^13^C) (ring-1,2-^13^C_2_, dicarboxyl-^13^C_2_, 99.0%) were obtained from Cambridge Isotope (Andover, MA, USA); β-glucuronidase *Escherichia coli* k-12 enzyme was from Roche (Manheim, Germany) and β-glucuronidase-arylsulfatase enzyme from *Elix pomatia* was provided by Sigma-Aldrich.

Glacial acetic acid (100% Merck, Darmstadt, Germany) was used for preparing the mobile phase and for Solid Phase Extraction (SPE), with purified water from a Milli-Q Plus system (Millipore, Milford, MA, USA).

The SPE OASIS HBL (6 cm^3^, 200 mg) cartridges were supplied by Waters (Massachusetts, Ireland) and Anotop 10LC syringe filter device (0.2 µm pore size, 10-mm diameter) was purchased from Whatman Inc. (Maidstone, UK). Acetonitrile and methanol were supplied by Carlo Erba Reagents S.r.l (MI, Italy). Synergy Polar-4u RP C-18 column (150 × 4.6 mm, 4 µm of particle size) was supplied by Phenomenex (Torrance, CA, USA). The urine samples were analysed on a Series 200LC quaternary pump (PerkinElmer, Norwalk, CT, USA), coupled with a AB/Sciex API 4000 triple-quadrupole mass spectrometry detector equipped with a Turbo Ion Spray (TIS) probe (Applied Biosystem, Warrington, Cheshire, UK) 

On the day of the clinical visit, each participant provided a spot urine sample, collected in a sterile polypropylene container and soon frozen at −20 °C until analysis.

The analytical equipment used for the urinary determination of metabolite levels was an HPLC/MS/MS. Seven phthalate metabolites (MEP; MBzP; MnBP; MEHP; MEHHP; MnOP; MiNP) were determined, using minor modifications of a previously published analytical method [54]. A molar weighted sum of DEHP metabolites was also evaluated for each sample.

With the same instrumental parameters, the BPA concentration was analysed in the urine samples, using a different pre- analytical treatment: samples were incubated at 38 °C for 2 h with β-glucuronidase-arylsolfatase enzyme from *Elix Pomatia*, and then 1mL of acetic acid 2% (*v*/*v*) and the deuterium labelled internal standard (BPA d_6_) were added. The solid phase extraction was subsequently carried out with SPE OASIS HBL (6 cm^3^, 200 mg) cartridge, previously conditioned with 3 mL of methanol, 3 mL of acetic acid 0.5% in water (*v*/*v*) and 2.5 mL of methanol.

The following *m*/*z* ion combinations (parent ion to daughter ion) were monitored in the negative ion mode: 227/212 for the native BPA and, 233/215 for the internal standard. The complete analysis time was 6 min. All results were normalized by the urine creatinine concentration, determined using the alkaline picrate test with UV/Vis detection at 490 nm [55].

### 2.3. Semen Collection and Analysis

All male patients were required to undergo 2–7 days of sexual abstinence before providing the semen samples. After liquefaction of the semen (about 30 min at 37 °C), the sperm samples were analysed for volume, concentration and motility, according to WHO criteria (2010) [56]. Semen volume was measured by weighing assuming a semen density of 1.0 g/mL. A hemocytometer (Bioanalytic GnbH, Umkirch, Germany) was used to estimate sperm concentration, with volume of semen being dispensed using Gilson Microman M25, M50 or M250 positive displacement pipettes (Gilson UK, Luton UK) as appropriate to the dilution being made. Motility was scored manually, as percentages of fast-forward progressive, slow forward progressive, non-progressive and immotile spermatozoa over 200 spermatozoa in at least five power fields per replicate.

The SEMinal QUAlity studies (SEMQUA) checklist was followed to improve accuracy and transparency of the study [57]. Both an internal and external quality control program, according to the guidelines of the European Society of Human Reproduction and Embryology (ESHRE) [58], has been established in the laboratory in order to control random and systematic errors and interlaboratory differences. All the laboratory personnel were trained according to the ESHRE Special Interest Group in Andrology Basic Semen Analysis Course.

### 2.4. Statistical Analysis

Descriptive statistic was conducted for the population characteristics, along with the distributions of urinary metabolites levels and semen quality parameters. The use of parametric or non-parametric methods, where appropriate, permitted to identify potential confounding factors and differences between groups, in demographic and habits categories. Furthermore, where possible, results were logarithmic transformed, to better adapt the data to the study design and to obtain a normal distribution.

Data of metabolite concentrations were divided into quartiles and therefore the distribution of semen parameters was studied. Moreover, we used a multivariate regression models to estimate the association between metabolite concentrations and semen quality. Tests for trend across quartiles in regression models were conducted. All regression analyses were achieved using data corrected for creatinine measurement. The statistical analysis was carried out with the software SPSS version 22 (IBM, Armonk, NY, USA) 

The three semen parameters are normally distributed so that for the statistical analysis the Analysis of Variance (ANOVA) test was used. A linear regression analysis was conducted between the semen parameters and the phthalate metabolites levels to evaluate any possible correlation. The ANOVA test was used to understand the main potential sources of xenoestrogens of interest in living environment (use of plastic containers for food storage; eating habits such as taking products containing phytoestrogens, such as soy, or use of canned food; use of enamels; use of hair sprays and perfumes).

Age, BMI, alcohol use and genital pathologies were considered as confounding factors, for their incidence on male fertility. In particular, for age, is documented in literature how semen volume has a mild reduction with time, semen concentration doesn’t change significantly with higher age while spermatozoa motility shows a defined decrease of about 0.2/0.6% per year [59].

## 3. Results

### 3.1. Characteristics of Study Population

Characteristics of the enrolled population are listed in Table 1.

### 3.2. Chemical Exposure and Semen Quality

The semen analysis results were: volume (mL) equal to 2.6 ± 0.6 (5th/95th percentile: 1.0/4.0); sperm concentration (expressed as a natural logarithm-Ln) equal to 3.26 ± 1.20 (5th/95th percentile: 0.22/4.58); motility (%) equal to 32.9 ± 17.3 (5th/95th percentile 3.6/60.0). According to the WHO reference values [56], 8 men (7.62%) had a low volume (<1.5 mL), 32 men (30.42%) had a low sperm concentration (<15 × 10^6^) and 64 men (60%) had a total motility of spermatozoa below 40%. Furthermore, 32.3% of the sample population had more than one abnormal semen parameter compared to the WHO reference values. In Table 2 more details about seminal fluid characteristics are presented. In Table 3 the analytical results regarding urinary metabolites of phthalates and BPA, corrected for creatinine levels, (µg/g creatinine) are presented.

**Figure 1:**
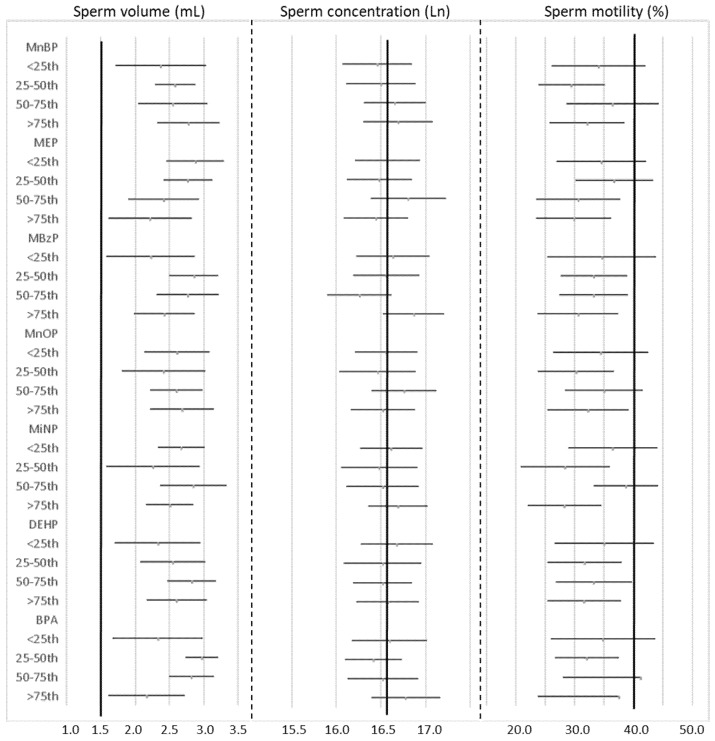
Average estimates and 95% confidence intervals of semen parameters in relationship with natural logarithm of metabolites concentration, from adjusted regression models, compared with the WHO reference values (1.5 mL for volume; 16.52 for Ln of sperm concentration; 40% for motility) represented by the vertical black lines.

Figure 1 presents a division into concentration quartiles, for each metabolite, to observe the trend of the various semen parameters. The aim was to highlight a possible modification of sperm volume, concentration or motility across the different quartiles. However, no statistically significant differences (ANOVA, *p* > 0.05) emerged.

Looking at the ranges of semen parameters in every quartile, the sperm volume was completely above the WHO “normal” value, the sperm concentration was around the limit, while the motility was the most critical element, with range mainly under the WHO reference value.

Table 4 shows the coefficients and corresponding 95% confidence intervals for the association between each semen parameter and each metabolite concentration (as natural logarithms) moreover the model was adjusted for confounding variables (BMI, age, smoking habits, drinking alcohol, male genital anomalies).

The regression study showed that the volume was positively associated with Ln(MnBP), Ln(MnOP) and Ln(BPA) levels while was negatively associated with Ln(MiNP) level.

**Figure 2:**
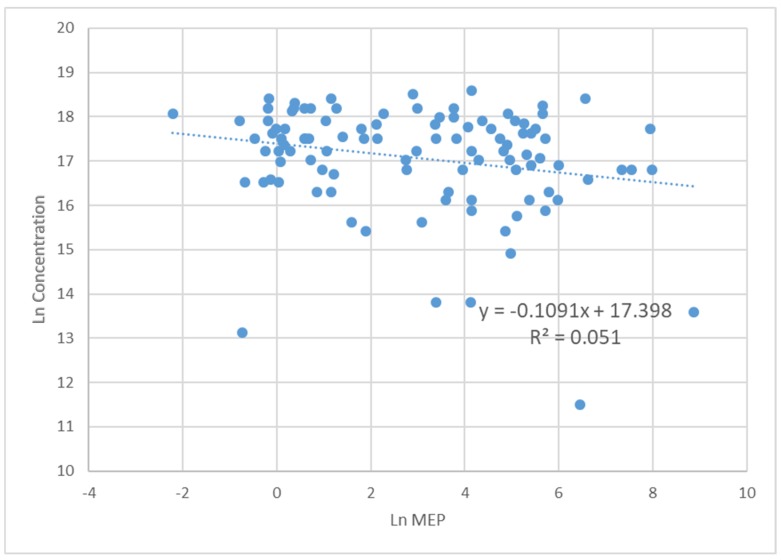
Scatterplot of semen concentration, as natural logarithm, vs Ln(MEP), with superimposed regression line from the covariate adjusted model.

In addition, the natural logarithm of the sperm concentration had a statistically significant inverse relationship with the Ln(MEP) level (Figure 2). For a sensitivity analysis we conducted a stratification by age, dividing the sample into two groups, over (43 subjects) and under (62 subjects) 40 years. Although the linear regression analysis, carried out separately in the two groups, re-proposed what emerged in the overall sample, some differences emerged. In the older group the significance between volume and Ln(MnBP) and Ln(BPA) was lost. In the younger group were maintained a positive association between volume and Ln(BPA) (β = 0.408, *p* = 0.023) and an inverse association between Ln(concentration) and Ln(MEP) (β = −0.332, *p* = 0.028); furthermore, a positive correlation between sperm concentration and Ln(MBzP) (β = 0.655, *p* = 0.023) and between motility and Ln(MBzP) (β = 0.609, *p* = 0.0.36) added.

A second stratification was made, dividing the sample between men with alteration of semen parameters (63 subjects) and men without any alteration (42 subjects). The linear regression analysis re-proposed the same correlations of the overall sample, moreover in the group with normal seminal fluid an inverse correlation emerged between the sperm concentration and the levels of BPA (β = −0.476, *p* = 0.006), previously not evidenced.

### 3.3. Study about Potential Sources of Exposure

A significant correlation emerged (*p* = 0.005) between the consumption of canned food and the levels of BPA (natural logarithm). Those who declared a daily consumption showed significantly higher values than those who reported that they never ate them (1.03 vs. −2.43 as average values, corresponding to 2.81 vs. 0.14 µg/g creat).

Another important and significant result (*p* = 0.00) was the correlation between habit of daily use of perfumes and significant higher urinary levels of MEP (as natural logarithm) compared to a less frequent use, and in particular the non-use (3.98 vs. 1.77 as average values, corresponding to 389.86 vs. 48.68 µg/g creat).

Table 5 presents the stratified data for work activity. Nevertheless, no statistically significant differences for the various analysed parameters were found (*p* > 0.05).

The statistical elaboration (Table 6) led us to highlight a significant negative association between the semen volume and the habit of using plastic containers for food storage (*p* = 0.037) with greater volume for subjects who declared that they never use plastic containers, compared to those who claimed to use it daily (3.06 vs. 2.30 mL as average values).

In conclusion, no further statistically significant associations were found for other metabolites or the parameters of the seminal fluid, in relation to the exposure sources considered.

## 4. Discussion

Endocrine disrupting pollution, particularly mediated by phthalates and alkyl phenols, represents a ubiquitous risk factor that might strongly affect global male reproductive health. The reproductive toxicity of these substances has emerged in both animal and cell models [60,61]. There is still debate about the possible mechanisms by which phthalates and/or BPA may damage the male reproductive system, but from the literature, multiple pathways (Nrf2-mediated Notch1 signaling [25], PTEN/AKN pathway [62], ERK1/2 pathway [26]) and multiple target cells might be involved [63]. A structure-activity relationship (SAR) study showed that phthalates with straight side chains (C_4_–C_6_) have higher male reproduction toxicity, as these phthalates down-regulate the insulin-like peptide 3 (INSL3), involved in the testis descent, and cytochrome P450 11A1 (CYp11a1), causing a lower testosterone production and subsequent reproductive tract anomalies. Furthermore, these compounds act negatively on testosterone production even directly inhibiting the androgen-biosynthetic enzyme activity and glucorticoid-metabolizing activity [64]. BPA, based on observed experimental evidences, has estrogenic and antiandrogenic effects, it produces an alteration of gonadotropin levels that induces a lower testosterone production by Leydig cells, and this hormone, converted to dihydrotestosterone, is responsible for spermatogenesis, transport and storage of spermatozoa [65].

The current study has confirmed some associations previously demonstrated by cross-sectional studies. MEP levels were found to be negatively associated with sperm concentration, in agreement with other reports from Sweden, Poland and India [66,67,68].

The inverse correlation between sperm concentration and MEP levels represents an interesting finding, since this phthalate is still widely used in everyday products and it is not subject to regulatory restrictions in Europe. Its toxicity to the endocrine system is well documented, even if with some uncertainties. In in vitro recombinant/receptor gene bioassay diethylphthalate (DEP) exhibited a weak activity with human estrogen receptor (hER) in human breast cancer cell line MCF-7 and in yeast cells [69]. In sperm suspensions from volunteers, the mean motility was shown to decrease when exposed in vitro to DEP (33, 330, 3300 µmol/L), with a time and dose-dependent action and an inhibition of 10% was seen at high dose [70].

The possibility of exposure to DEP due to perfume use is emerging. In the last few years, the use of phthalates in perfumes has gained attention. A characterization of 47 branded perfumes showed the presence of DEP in all the samples, with a mean value of 1621.6 ppm and a maximum of 23,649.2 ppm [71]. These results showed the absence of any industrial restriction, in fact, presently, the health risks for humans are not so clear. A study focused on a female population confirming that subjects reporting the use of perfumes had 2.9 times higher concentration of MEP than other women [72].

A correlation between sperm volume and several phthalates metabolites was reported in the literature with different results [13,17,20]. Semen volume reflects the excreting function of the accessory glands (seminal vesicles, prostate gland and bulbourethral glands), therefore a positive association (like for MiNP level in the present study) or a negative association (like for MnBP and MnOP levels) may indicate a change in the accessory gland function with mechanisms to be yet clarified. Indeed, the way in which an exposure to plasticizers can affect the produced spermatic volume is not clear and requires more experimental investigations.

The use of plastic containers for food storage, particularly for fatty ones, can lead to a migration of plasticizer molecules from the container to the food [73,74,75]. Although a specific contamination is not yet identified, the frequent use of plastic containers can determine a cumulative contribution of endocrine disruptors in the subjects’ feeding. The emerged finding, a link with decreased sperm volume, could indicate a possible source of exposure risk of some significance.

Data about BPA and canned food consumption are in line with previous published studies [76,77,78], BPA concentration after meal with canned food was higher, respectively, of 152% (after 2 h), 206% (after 4 h) 79% (after 6 h) [79]. The present results confirmed the ingestion as one of the major source of exposure to BPA in daily life. Nevertheless, the presented study did not highlight any statistically significant link between the urinary levels of BPA and alterations of the parameters of seminal fluid, as conversely documented in various literature studies. This could be due to the particularly reduced levels of BPA herein recorded, on average 0.24 ± 0.43 µg/g creatinine, compared to those identified in the literature with values two [80] to ten [34] or twenty times higher [40].

Our results are significantly higher than the most recent Italian reference values for phthalates [39], expressed in µg/g creatinine, for MnBP (Reference value 95th percentile (RV_95_) 4.8 vs 31.4 mean), MBzP (RV_95_ 3.0 vs. 4.4 mean) and molar sum of DEHP metabolites (RV_95_ 20.21 vs. 82.2). This difference is particularly important for the MnBP, being our mean value about eight time the reference value. The comparison with other published study presents lower differences but levels were determined in other geographical area, from five to twenty years ago and a comparison would be less accurate [39].

For what concerns BPA the only Italian reference value was produced in 2010 [81] with a RV_95_ of 13.0 µg/L that compared with the present result (1.22 µg/g creatinine) shows greater levels (even if the correct data for creatinine values is not presented in the paper of Galloway et al. [81], considering the maximum creatinine value for the normal range, the RV_95_ is confirmed greater), probably due to 9 years of European legislation aimed at the elimination and replacement of BPA-containing plastics.

The current study allows us to highlight an exposure to plasticizing chemicals, greater in a sample of hypofertile subjects compared to the general population. Further studies may characterize the causal elements but from this survey, the need to investigate life habits is clear. In view of the documented molecular effects of these substances, the possibility of affecting male reproductive health, and the quality of the seminal fluid in particular, seems to be concrete.

## 5. Conclusions

The added value of the present investigation to the ongoing scientific debate regarding the effects on male reproductive health due to plasticizers consists, as far as we know, in being the first study of this type in Italy. The geographical element plays an important role in environmental exposure studies, as demonstrated in the comparison of literature data in the general population [82], this study can be a starting point for future investigations in Italy. Secondly, the will to investigate possible sources of exposure and, thus, identify the possible causes, both in the workplace and in life habits, represents a deepening that is not easily found in cohort studies like this. To identify elements of life habits significantly involved in phthalates and/or BPA exposure, it’s an interesting outcome for future studies, both as a source of possible health risks and as confounding factors in studies aimed at assessing possible occupational exposures, for example.

We found a significant correlation between a lower sperm concentration and MEP urinary levels; this is a remarkable finding that supports a possible new risk factor for male reproduction.

Currently, DEP is not included in restriction or authorization regulations, as human data are still considered limited, compared to experimental data on the animal models. Nevertheless, the data emerging from recent surveys, such as the one presented herein, require greater attention specifically about the exposure levels detected with urine analysis.

Results help to identify two possible risk sources. The use of perfumes could represent a significant source of DEP, with a substantial increased MEP urinary levels in daily users; the consumption of canned food could represent a significant source of exposure to BPA, as already highlighted in the scientific literature [76].

Finally, the significant negative association between the semen volume and the habit of using plastic containers for food storage deserves to be better investigated, for the possible presence of different chemicals with additive or even synergistic effects at low doses.

The current study is a cross-sectional in design, a kind of study that can only allow the formulation of hypotheses but it cannot give risk indices or define properly causal links, due to the nature of the epidemiological investigation. Subsequent case control investigations, or prospective cohort study, will be necessary to confirm the correlation hypotheses highlighted in the present investigation.

Limitations of the present study are represented by the use of a single spot urine sample for the determination of the metabolites of interest, and of a single seminal liquid sample to determine its characteristics. However, some authors [83] have shown that repeated urinary samples from the same person tend to have metabolites distributed in the same quartile and concerning semen [84] the use of models with adjustments for covariates determined two samples that do not differ significantly from each other.

Three take home messages arise from this study:

First for stakeholders: different toxic agents can affect male reproductive health, and some of these are in daily life habits. Exposure to plasticizers could be achieved mainly through skin contact with cosmetics and perfumes and by ingestion, in the case of canned products or use of plastic containers for food. The control and elimination of possible avoidable sources of risk could be a way to improve your heath.

Second for the general population: promoting reproductive health involves right food choices and daily actions, to avoid possible unsafe exposures.

Third for peers: the identification of possible sources of exposure is a relevant datum to investigate, in order to be able to propose concrete actions for health protection, first in workplaces, asking about the specific tasks and risks of chemical exposure and then about daily life habits.

Some evidence emerged about the possible link between exposure to phthalates and/or BPA and seminal fluid quality; further case/control studies, and perspective studies, will allow us to confirm this causality hypothesis.

## Figures and Tables

**Table 1:** Characteristics of the study population.

Category	Subjects (*n* = 105)
Age (range)	40.5 (29–67)
BMI ^1^ (% of subjects in the class)	data
Normal	50.5
Overweight	41.9
Obese	4.8
Missing	2.69
Actually smokers (%)	27.6
Previously smokers (%)	29.5
Area of residence (%)	
Urban	74.3
Rural	14.3
Coast	4.8
Industrial	1.0
Urban and coast	1.0
Other	1.9
Missing	2.9
Use of plastic containers for fat food storage (%)	
Never	15.2
Daily	17.1
Weekly	45.7
Monthly	17.1
Missing	4.8
Eating canned food at least weekly (%)	49.6
Eating soya products at least weekly (%)	20.0
Use of scents at least weekly (%)	70.5
Use of hair sprays at least weekly (%)	10.5
Working activity (%)	
Employees, professionals, computer operators, drivers (prolonged sitting positions–exposure to heat in the genital area)	48.6
Farmers, artisans, dental technicians (chemicals)	17.1
Health workers (radiation, chemicals, night job…)	2.9
Armed forces	8.6
Other	14.3
Missing	8.6

^1^ BMI–body mass index.

**Table 2:** Analytical results of seminal liquid parameters.

Parameters	Volume (mL)	Sperm Concentration (Ln)	Total Motility (%)
Arithmetic mean	2.6	3.26	32.9
Standard deviation	0.9	1.20	17.3
Median	2.5	3.55	35.0
Range (min/max)	0.5/6.0	−2.30/4.79	0.0/90.0
5th percentile	1.0	0.22	3.6
95th percentile	4.0	4.58	60.0
% data <WHO ^1^ reference value	7.62	30.42	60.0

^1^ WHO–World Health Organization.

**Table 3:** Analytical results of urinary metabolites of phthalates and BPA, corrected for creatinine levels (µg/g creatinine).

Parameters	MnBP	MEP	MBzP	MnOP	MiNP	∑DEHP	BPA
Arithmetic mean	31.4	243	4.4	1.9	2.41	20.21	0.24
Standard deviation	36.5	821	4.2	3.1	7.01	31.18	0.43
Median	19.3	29	3.2	1.2	1.03	10.59	0.10
Range (min/max)	2.7/245.13	0/7116	0.4/28.5	0.0/20.9	0.01/63.92	1.79/205.01	0.02/2.81
5th percentile	4.5	1	1.1	0.0	0.01	2.56	0.03
95th percentile	112.4	1297	12.5	7.1	8.77	84.06	1.22
LOD ^1^ (µg/L)	0.8	3	1.2	0.1	0.02	0.15 (MEHP) 0.05(MEHHP)	0.02
% data > LOD	100	65	98	85	84	100	54

^1^ LOD–Limit of Detection.

**Table 4:** Estimated coefficients (95% confidence intervals) from separate multivariate adjusted ^1^ linear regression models of each semen parameters on the natural logarithm of each metabolite concentration.

Ln (Concentration)	Semen Volume			Semen Concentration (Ln)			Spermatozoa Motility		
	β ^2^	95% CI	*p* Value	β	95% CI	*p* Value	β	95% CI	*p* Value
MnBP	0.346	0.011/0.673	0.043	0.090	−0.309/0.547	0.582	0.050	−5.354/7.244	0.766
MEP	−0.107	−0.119/0.045	0.369	−0.303	−0.246/−0.035	0.010	−0.126	−2.388/0.716	0.287
MBzP	−0.053	−0.450/0.323	0.745	0.220	−0.148/0.851	0.166	0.087	−5.371/9.333	0.593
MnOP	0.875	0.007/0.968	0.047	−0.076	−0.678/0.565	0.857	0.522	−3.595/14.703	0.231
MiNP	−0.844	−0.720/−0.001	0.049	0.068	−0.426/0.504	0.869	−0.488	−10.827/2.858	0.250
molar sum DEHP	−0.355	−0.653/0.008	0.056	0.004	−0.433/0.422	0.981	−0.252	−10.661/1.920	0.171
BPA	0.296	0.044/0.452	0.018	−0.181	−0.467/0.061	0.129	0.035	−3.326/4.440	0.776

^1^ adjustment for confounding factors (age, smoke, BMI, alcohol use, genital pathologies), ^2^ β: Standardized regression coefficient

**Table 5:** Data stratification for job classes.

Job Classes	Mean Values with Standard Deviations									
	Semen Volume (mL)	Semen Concentration (Ln)	Spermatozoa Motility (%)	MnBP (Ln)	MEP (Ln)	MBzP (Ln)	MnOP (Ln)	MiNP (Ln)	Molar Sum DEHP (Ln)	BPA (Ln)
Employees, computer operators, drivers	2.56 ± 0.96	17.33 ± 0.90	37.02 ± 16.52	3.04 ± 0.86	3.21 ± 2.57	1.18 ± 0.72	−0.32 ± 1.47	−0.68 ± 1.98	2.40 ± 0.81	−2.02 ± 1.08
Farmers, artisans, dental technicians	2.82 ± 1.17	16.92 ± 1.40	30.12 ± 16.36	3.03 ± 0.85	2.05 ± 2.24	1.24 ± 0.64	0.39 ± 1.68	0.12 ± 2.04	2.73 ± 1.08	−2.37 ± 0.67
Health workers	2.33 ± 0.76	16.44 ± 1.36	22.67 ± 18.61	3.13 ± 1.44	5.24 ± 0.66	0.82 ± 1.69	0.23 ± 1.99	0.58 ± 2.70	2.78 ± 2.01	−1.52 ± 1.42
Armed forces	2.50 ± 0.61	16.89 ± 0.77	30.56 ± 17.40	3.40 ± 1.13	3.43 ± 1.66	1.38 ± 0.59	0.27 ± 1.07	0.01 ± 1.37	2.51 ± 0.75	−2.21 ± 1.08
Other	2.93 ± 0.62	17.14 ± 0.94	36.79 ± 16.01	2.89 ± 0.80	2.79 ± 2.48	1.17 ± 0.86	−0.86 ± 1.54	−1.46 ± 2.01	2.30 ± 1.11	−1.66 ± 1.22
P value ANOVA test	0.759	0.449	0.363	0.853	0.184	0.834	0.239	0.231	0.741	0.430

**Table 6:** Data stratification for food/life habits (mean values with standard deviations).

Parameters under Consideration	Semen Volume (mL)	Semen Concentration (Ln)	Spermatozoa Motility (%)	MnBP (Ln)	MEP (Ln)	MBzP (Ln)	MnOP (Ln)	MiNP (Ln)	Molar Sum DEHP (Ln)	BPA (Ln)
Use of plastic containers for food storage									
Never	3.06 ± 1.06	17.13 ± 1.13	29.69 ± 12.31	3.29 ± 0.56	3.13 ± 1.70	1.18 ± 0.65	−0.50 ± 1.81	−0.94 ± 2.40	2.59 ± 0.99	−2.29 ± 0.84
Daily	2.30 ± 0.79	17.06 ± 0.82	29.11 ± 14.24	2.98 ± 1.00	2.84 ± 2.66	1.31 ± 1.02	0.01 ± 1.60	−0.28 ± 1.95	2.51 ± 1.16	−2.17 ± 1.20
Weekly	2.73 ± 0.86	17.13 ± 1.31	37.84 ± 18.53	3.04 ± 1.03	2.85 ± 2.63	1.10 ± 0.76	−0.30 ± 1.51	−0.58 ± 2.11	2.42 ± 0.91	−2.03 ± 1.15
Monthly	2.39 ± 0.88	16.86 ± 1.28	29.47 ± 18.95	2.87 ± 0.69	3.06 ± 3.09	1.23 ± 0.65	−0.22 ± 1.18	−0.37 ± 1.65	2.22 ± 0.76	−1.96 ± 0.82
*p* value ANOVA test	0.037	0.653	0.058	0.523	0.99	0.857	0.817	0.834	0.952	0.752
Eating canned food										
Never	2.70 ± 1.15	17.16 ± 1.31	31.13 ± 14.98	2.82 ± 0.74	2.51 ± 2.51	1.05 ± 1.05	−0.39 ± 1.54	−0.74 ± 1.93	2.34 ± 1.19	−2.43 ± 0.79
Daily ^1^	4	16.81	60	4.44	3.95	1.76	−3.22	-4.61	3.47	1.03
Weekly	2.63 ± 0.86	16.92 ± 1.33	32.19 ± 17.23	2.99 ± 0.91	2.92 ± 2.60	1.13 ± 0.66	−0.09 ± 1.34	−0.29 ± 1.80	2.30 ± 0.82	−2.21 ± 0.95
Monthly	2.58 ± 0.88	17.26 ± 0.86	35.42 ± 17.98	3.18 ± 3.18	3.12 ± 2.68	1.28 ± 0.87	−0.36 ± 1.71	−0.70 ± 2.36	2.64 ± 0.98	−1.85 ± 1.14
*p* value ANOVA test	0.372	0.923	0.433	0.251	0.852	0.731	0.216	0.16	0.188	0.005
Eating soya products										
Never	2.65 ± 1.06	17.18 ± 0.89	32.34 ± 16.30	2.88 ± 2.88	2.33 ± 2.33	1.03 ± 0.73	−0.29 ± 1.40	−0.52 ± 1.92	2.30 ± 0.76	−2.19 ± 1.02
Daily	3.75 ± 0.35	17.66 ± 1.07	40.00 ± 0.00	3.16 ± 0.94	3.28 ± 3.01	1.57 ± 0.40	1.91 ± 0.67	1.21 ± 0.13	3.17 ± 0.55	−1.44 ± 1.36
Weekly	2.67 ± 0.73	16.84 ± 1.57	36.39 ± 19.01	3.10 ± 0.90	3.67 ± 2.88	1.47 ± 0.92	−0.72 ± 1.55	−1.27 ± 2.10	2.45 ± 1.14	−2.16 ± 1.12
Monthly	2.55 ± 0.80	17.03 ± 1.29	32.66 ± 18.04	3.22 ± 0.92	3.27 ± 2.54	1.16 ± 0.73	−0.11 ± 1.60	−0.29 ± 2.17	2.54 ± 1.05	−1.97 ± 1.08
*p* value ANOVA test	0.384	0.641	0.811	0.435	0.266	0.208	0.106	0.236	0.379	0.629
Use of scents										
Never	2.88 ± 1.06	17.09 ± 1.20	32.86 ± 19.13	2.86 ± 0.85	1.77 ± 2.10	0.85 ± 0.69	−0.62 ± 1.52	−0.93 ± 2.07	2.07 ± 0.84	−2.15 ± 1.21
Daily	2.57 ± 0.90	16.98 ± 1.30	34.14 ± 17.78	3.20 ± 0.95	3.98 ± 2.41	1.30 ± 0.84	−0.34 ± 1.54	−0.64 ± 2.11	2.55 ± 0.96	−2.08 ± 1.10
Weekly	2.53 ± 0.74	17.45 ± 0.66	34.33 ± 13.61	2.79 ± 0.90	1.78 ± 2.45	1.21 ± 0.64	0.03 ± 1.51	−0.21 ± 1.99	2.42 ± 0.99	−2.06 ± 0.63
Monthly	2.58 ± 0.97	16.88 ± 1.10	26.67 ± 15.06	2.96 ± 0.62	0.96 ± 1.13	1.13 ± 0.63	0.94 ± 0.85	0.76 ± 1.20	2.75 ± 0.89	−2.06 ± 1.22
*p* value ANOVA test	0.747	0.893	0.736	0.329	0	0.203	0.117	0.295	0.24	0.961
Use of hair spray										
Never	2.65 ± 0.93	17.01 ± 1.23	32.44 ± 17.20	3.05 ± 0.86	2.86 ± 2.52	1.16 ± 0.79	−0.22 ± 1.51	−0.48 ± 2.02	2.40 ± 0.89	−2.14 ± 1.11
Daily	2.67 ± 0.94	17.53 ± 0.81	34.00 ± 17.90	3.01 ± 1.18	2.62 ± 3.01	1.16 ± 0.62	−0.67 ± 1.87	−1.01 ± 2.66	2.50 ± 1.21	−1.89 ± 0.66
Weekly	2.00 ± 0.71	18.02 ± 0.25	50.00 ± 0.00	2.87 ± 0.16	2.82 ± 3.46	1.43 ± 0.37	0.40 ± 0.54	0.39 ± 0.27	2.56 ± 0.44	−1.61 ± 0.3
Monthly	2.75 ± 0.87	16.91 ± 0.99	42.50 ± 17.08	3.03 ± 1.64	5.12 ± 0.76	1.26 ± 1.16	−0.45 ± 1.45	−1.23 ± 1.77	2.84 ± 1.66	−1.83 ± 1.04
*p* value ANOVA test	0.811	0.39	0.342	0.995	0.368	0.966	0.794	0.745	0.821	0.813

^1^ one person.
